# Impact of the COVID-19 Pandemic on Community Consumption of Antibiotics for Systemic Use and Resistance of Invasive *Streptococcus pneumoniae* in Slovenia

**DOI:** 10.3390/antibiotics12060945

**Published:** 2023-05-23

**Authors:** Tamara Kastrin, Verica Mioč, Aleksander Mahnič, Milan Čižman, Slovenian Meningitidis Study Group

**Affiliations:** 1 Department for Public Health Microbiology, National Laboratory of Health, Environment and Food, 1000 Ljubljana, Slovenia; verica.mioc@nlzoh.si; 2 Department for Microbiological Research, National Laboratory of Health, Environment and Food, 2000 Maribor, Slovenia; aleksander.mahnic@nlzoh.si; 3 Department of Infectious Diseases, University Medical Centre, 1000 Ljubljana, Slovenia

**Keywords:** resistance, antibiotic, consumption, *S. pneumoniae*, serotype, Slovenia

## Abstract

The present study aims to investigate the impact of the COVID-19 pandemic on community antibiotic consumption and the resistance of invasive *Streptococcus pneumoniae* (2015–2022) to penicillin in Slovenia. During the pandemic in 2020 and 2021, the total use of antibiotics for systemic use decreased by 23.5% and 24.3%, expressed in defined daily doses per 1000 inhabitants per day (DID), while the use of penicillins, macrolides and broad-spectrum penicillins decreased by 30%, 20% and by 17.5%, respectively, and that of broad-spectrum macrolides fell by 17.1%. The incidence of invasive pneumococcal diseases (IPD) in Slovenia had a large decline during the pandemic. Decreased resistance to macrolides was significantly associated with decreased use of macrolides, while for penicillins the correlation could not be statistically confirmed. The proportion of PCV13 serotypes in IPD in Slovenia decreased after the introduction of the vaccine in the national programme, falling from 81.6% in 2015 to 45.5% in 2022. We noticed a decrease in the serotypes 1, 14, 9V, 7F, 4, 6A and an increase in the serotypes 3, 8, 22F, 11A, 23A and 15A. National interventions during the COVID-19 pandemic substantially decreased outpatients’ antibiotic consumption, as well as incidence and resistance of invasive *S. pneumoniae.*

## 1. Introduction

Since its start in early 2020, the COVID-19 pandemic has had a substantial impact on health systems worldwide. The pandemic also had significant effects on the prescription of antibiotics. In many high-income countries, antibiotic use fell significantly in 2020 compared to 2019, due to the reduced use of outpatient health care services and a fall in the spread of other respiratory illnesses that are generally treated with antibiotics [1,2,3,4,5]. In the community, the EU/EEA population-weighted mean declined from 18.3 [8.7–32.4] defined daily doses (DDD) per 1000 inhabitants per day (DID) in 2019 to 15.0 [7.1–26.4] DID in 2020, representing a 18.3% decrease [3]. However, it is still unclear whether this reduced community antibiotic consumption was sustained throughout 2021, and what implications it may have for antibiotic resistance. Antibiotic use is a known risk factor for the emergence of antibiotic resistance, but demonstrating a causal relationship between antibiotic use and resistance is challenging. Several studies have found associations between total antibiotic and penicillin use with regard to the resistance of *Streptococcus pneumoniae* to penicillin [6,7,8,9]. In contrast, a study published by Olesen et al. showed weak or no correlations between total antibiotic use and the use of beta-lactams with regard to the resistance of *S. pneumoniae* [10]. However, Goossens H et al. showed that macrolide use is the single most important driver of the emergence of macrolide resistance in vivo [11]. If the use of antimicrobials is the main driver of resistance, it seems logical to reduce resistance by reducing the use of them. Still, the evidence for positive correlations is mixed [12,13,14,15,16]. In our study from 1997 to 2017, we observed an increase in macrolide resistance among invasive *S. pneumoniae* in Slovenia, despite reduced macrolide consumption. We demonstrated that the cause was the spread of the England^14^-9 clonal cluster [16].

Slovenia introduced vaccination with the 7-valent pneumococcal conjugated vaccine (PCV-7) for children with high-risk conditions in 2006. In 2009 and 2010, PCV-10 and PCV-13 replaced PCV-7, respectively. In 2012, PCV-13 was also made available for vaccination of adults with high-risk conditions. In 2015, PCV-10 was introduced in the national vaccine recommendations programme, and in 2019 it was replaced by PCV-13. In the period 2015–2019, moderate coverage (49–65%) was achieved. In 2020, the vaccine coverage increased to 70%, while in 2021 it fell to 58% [17].

The present study aims to investigate the impact of the COVID-19 pandemic on community antibiotic consumption in 2020–2021 compared with the period 2015–2019 and the resistance of invasive *S. pneumoniae* isolates in Slovenia in the period 2015–2022, as well as to investigate whether there is any correlation between antibiotic use and resistance in this context. Understanding the relationship between use and resistance is important, because it allows accurate predictions of the future of antibiotic resistance and enables the development of a better goal-oriented antibiotic stewardship policy.

## 2. Results

### 2.1. Consumption of Antibiotics

During the pre-COVID-19 period, the total consumption of antibiotics for systemic use (J01) in Slovenia was stable, between 11.9% and 11.5% DID and between 524 and 470 prescriptions per 1000 inhabitants per year (RxIDs), respectively (Table 1) [18,19]. During the COVID-19 pandemic, in 2020 and 2021, the total use of J01 decreased by 23.5% and 24.3% as expressed in DID, respectively. The use of penicillins (J01C), macrolides (J01FA) and broad-spectrum penicillins (J01CR) decreased by 30%, 20% and 17.5% as expressed in DID, and by 30.8%, 24.7% and 10.2% expressed in RxIDs. The consumption of broad-spectrum macrolides (J01FA minus J01FA1) according to the ECDC classification decreased by 17.1% as expressed in DID and 21.6% in RxIDs [20].

### 2.2. Incidence of Invasive Streptococcus pneumoniae in Slovenia

**Figure 1:**
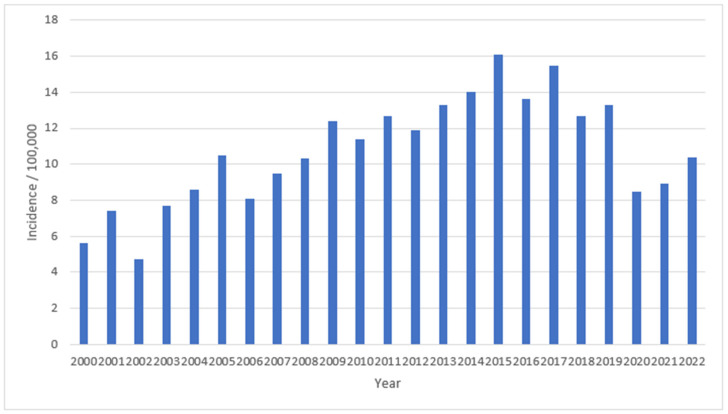
Incidence of invasive *Streptococcus pneumoniae* in Slovenia, 2000–2022.

As seen in Figure 1, the overall incidence of invasive *S. pneumoniae* in Slovenia appears to have increased steadily from 5.6/100,000 in 2000 to 16.1/100,000 in 2015, followed by a downward trend and a large decline during the COVID-19 pandemic (8.5/100,000 in 2020 and 8.9/100,000 in 2021). Overall, the incidence of invasive *S. pneumoniae* in Slovenia has decreased significantly since 2015 (Pearson’s r = −0.856, *p* = 0.007).

### 2.3. Resistance of Invasive Streptococcus pneumoniae to Penicillin and Macrolides

The resistance of invasive *S. pneumoniae* to penicillin and macrolides is shown in Table 2. In the pre-COVID-19 period, the resistance to penicillin was between 7.9% and 12.3%. In the first year of COVID-19, this resistance increased by 18.6% to 14.6%, but in the second and third years it decreased by 56.8% and by 53.5% to 6.3% and 6.8%, respectively. The resistance to macrolides decreased, in the pre-COVID-19 period from 2015 to 2019, by 47.3%, from 18.8% to 9.9%. In the first year of COVID-19, 2020, the macrolide resistance increased by 36.3% to 13.5%, and then decreased by 45.2% and by 40.8% to 7.4% and 8.0% in 2021 and 2022, respectively.

Among the 201 (9.6%) isolates less susceptible to penicillin from 2015 to 2022, 18 different serotypes were determined. Serotype 19A was the most prevalent (*n* = 64; 31.8%), followed by serotypes 14 (*n* = 39; 19.4%), 19F (*n* = 21; 10.4%), 9V (*n* = 17; 8.5%), and 6A (*n* = 11; 5.5%), while other serotypes were less represented. Among the 262 (12.6%) isolates less susceptible to erythromycin, 19 different serotypes were determined. Serotype 14 was the most prevalent (*n* = 112; 42.7%), followed by 19A (*n* = 42; 16.0%), 19F (*n* = 36; 13.7%), 6A (*n* = 11; 4.2%) 9V (*n* = 10; 3.8%), while other serotypes were less represented. Co-resistance to penicillin and erythromycin was present in 101 (4.8%) isolates. Serotype 19A was the most prevalent (*n* = 36), followed by 19F (*n* = 15), 6A and 9V (*n* = 9), 14 and 15A (*n* = 6), and some other serotypes, which were less represented.

The most frequent co-resistance to oral antibiotics used in outpatients was resistance to erythromycin and penicillin (*n* = 101; 50.2%), penicillin and tetracycline (*n* = 98; 48.8%) and penicillin and co-trimoxazol (*n* = 89; 44.3%). Among the combinations of co-resistance to penicillin, penicillin plus co-trimoxazol was the most prevalent (*n* = 18; 9.0%), followed by penicillin plus ampicillin, cefuroxime, erythromycin, and tetracycline (*n* = 16; 8.0%) and the combination of penicillin, erythromycin and tetracycline (*n* = 16; 8.0%).

### 2.4. Correlation between Consumption of Total Antibiotics for Systemic Use, Penicillins, Broad-Spectrum Penicillins, Macrolides and Broad-Spectrum Macrolides and Resistance of Invasive S. pneumoniae to Penicillin (Intermediate and Resistant) and Macrolides

**Figure 2:**
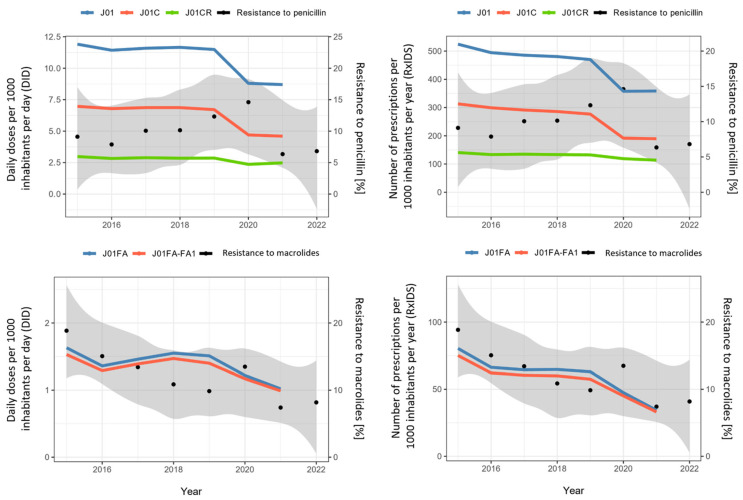
Correlations between the total use of antibiotics for systemic use (J01), penicillins (J01C), broad-spectrum penicillins (J01CR), macrolides (J01FA) and broad-spectrum macrolides (J01FA-FA1) expressed in DID and in RxIDs and the resistance of invasive strains of *S. pneumoniae* in Slovenia 2015–2022.

No correlation was found between the total antibiotic consumption for systemic use (J01), consumption of penicillins (J01C, J01CR) and resistance to penicillin in *S. pneumoniae* (Figure 2, top panels). A comparable trend over a longer period would be required to statistically confirm the correlation. The resistance to macrolides was significantly correlated with total use of macrolides (J01FA), as expressed in DID (Spearman’s rho2 = 0.731, *p* = 0.040) and RxIDs (Spearman’s rho2 = 0.786, *p* = 0.028,) and the use of broad-spectrum macrolides (Spearman’s rho2 = 0.810, *p* = 0.022) (Figure 2, bottom panels).

### 2.5. Serotype Distribution in Invasive S. pneumoniae in Slovenia

**Figure 3:**
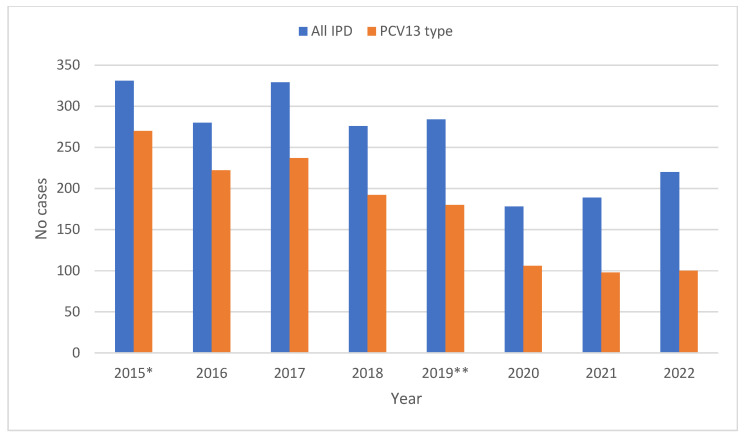
Yearly distribution of PCV13 and non-PCV13 serotypes among IPD in Slovenia, 2015 to 2022, * PCV10 introduced, ** PCV13 introduced.

During the period 2015 to 2022, we received 2087 IPD isolates. The most common serotype was serotype 3 (*n* = 373, 17.9%), followed by serotypes 14 (*n* = 206, 9.9%), 19A (*n* = 141, 6.8%), 1 (*n* = 130, 6.2%), 8 (*n* = 113, 5.4%), 9V (*n* = 111, 5.3%), 7F (*n* = 103, 5.0%), 4 (*n* = 101, 4.8%), and 22F (*n* = 80, 3.8%), while other serotypes were represented in lower proportions. The proportion of PCV13 serotypes in Slovenia decreased after the introduction of the pneumococcal conjugate vaccine in the national programme in 2015, from 81.6% in 2015 to 45.5% in 2022 (Figure 3).

**Figure 4:**
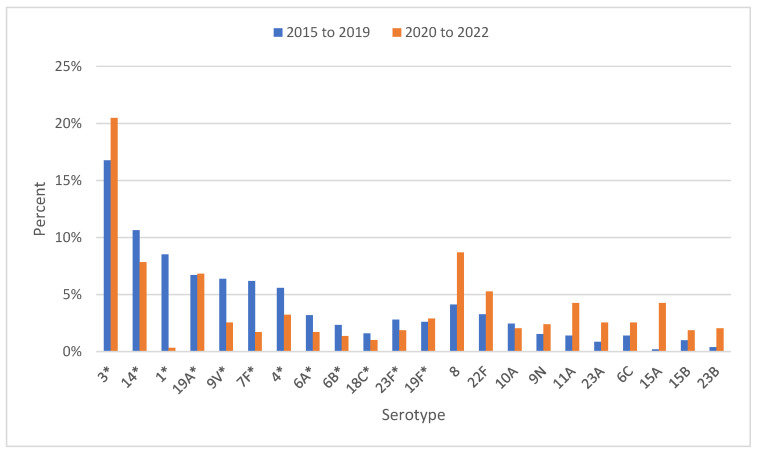
Proportions of the most common serotypes of IPD in Slovenia in two periods, 2015–2019 and 2020–2022, * PCV13 serotype.

In comparing the pre-COVID-19 period (2015–2019) to the COVID-19 period (2020–2022), we noticed a decrease in serotypes 1, 14, 9V, 7F, 4, 6A in the latter, and an increase in serotypes 8, 22F, 11A, 6C, 23A, 15A, as well as PCV13 vaccine serotype 3 (Figure 4). However, we must be cautious when interpreting the data on the proportions, as the total numbers of isolates is much lower in the COVID-19 period (2020–2022).

## 3. Discussion

The results presented above show that the total use of antibiotics in the community decreased in 2020 compared with 2019 and was maintained at about the same level in 2021, as measured in DID and RxIDs. The decrease was largest since 1996. Although similar changes (16% to 31%) in community antibiotic consumption have been described at the local and national levels, the observation period was a maximum of one year in these earlier studies [1,2,3,4,5]. In the first year of the COVID-19 pandemic, 2020, the resistance of invasive *S. pneumoniae* to penicillin increased by 18.6% to 14.6%, but in the second and third years it decreased by 56.8% and by 53.5% to 6.3% and 6.8%, respectively. The resistance to macrolides decreased in the pre-COVID-19 period from 2015 to 2019 by 47.3%, from 18.8% to 9.9%. In the first year of COVID-19, 2020, the macrolide resistance increased by 36.3% to 13.5%, and then decreased by 45.2% and by 40.8% to 7.4% and 8.0% in 2021 and 2022, respectively. In the period 2019–2021, in the EU/EEA countries, the mean community consumption of antibiotics for systemic use declined by 18.1% from 18.3 DID to 15.0 DID, and a mean increase in resistance to penicillin (33%) and macrolides (26.2%) was identified [20,21,22]. Penicillin resistance increased from a mean of 12.2% (range 4.0–33.3%) to 16.3% (range 3.6–35.7%) and macrolide resistance from a mean of 14.5% (range 3.5–30.4%) to 18.3% (range 0.0–36.0%) [22]. The consumption of outpatients antibiotics and resistance of *S. pneumoniae* in Sweden, Denmark, The Netherlands and Slovenia is shown on Table 3 [23,24,25,26,27]. The consumption of antibiotics for systemic use and macrolides decreased in all four countries but the resistance to penicillin increased in two and to macrolides in one country. These data show that reduced consumption of antibiotics is not enough to reverse antibiotic resistance in the community and that the relationship between antibiotic use and resistance is a complex issue [12].

The consumption of penicillins, the combination of penicillin with a beta-lactamase inhibitor (in our case only co-amoxiclav), macrolides and broad-spectrum macrolides expressed in DID and RxIDs also decreased substantially (Table 1). No correlation was found between the total antibiotic consumption for systemic use, consumption of penicillins and resistance to penicillin in invasive *S. pneumoniae*, despite the marked decrease in consumption. However, antibiotic resistance is a complex, temporally dynamic phenomenon, and many factors complicate the use–resistance association, making what should be an “obvious” connection sometimes difficult to identify and quantify [28]. In contrast, a high correlation between the use of macrolides and broad-spectrum macrolides and the resistance of *S. pneumoniae* to macrolides was indicated, completely different from what we found in our earlier study [16]. The correlation between the use of macrolides and the resistance of pneumococci to macrolides was reported previously, but no study that we are aware of reports a correlation between the use of broad-spectrum macrolides and resistance to macrolides [7,8,9,14,15]. In our previous study, we demonstrated the occurrence of a genetically related strain of *S. pneumoniae* of serotype 14, which was the cause of high macrolide resistance among invasive pneumococci in Slovenia, with the peak in 2011 [16]. We can now assume that the introduction of vaccination had an impact on this clone, as it is a vaccine serotype 14, and consequently on the reduction in macrolide resistance. We have also observed the decrease in other vaccine serotypes, which is, in addition to the downward trend in the incidence of IPD, further evidence in favour of the introduction of pneumococcal vaccination in the national vaccine scheme in 2015.

In contrast to our observations, an increase in the proportion of pneumococcal strains with reduced susceptibility to β-lactams and erythromycin was observed in a Spanish national study in 2020, coinciding with the emergence of SARS-CoV-2 [29]. Serotypes included in PCV7 and PCV13 showed a decline after the introduction of PCVs in Spain (PCV13 was included in national immunisation schedule in 2016). However, an increase in non-PCV13 serotypes (mainly 11A, 24F and 23B) that were not susceptible to penicillin promptly appeared. The use of antibiotics to prevent co-infections in patients with COVID-19 might have affected the increased proportion of pneumococcal-resistant strains in Spain. Slovenia has long-term IPD surveillance, and there has been a constant increase in the incidence of IPD since the beginning of this surveillance in 1993 to 2015, when it reached 16.1/100,000, followed by a downward trend and later a large decline during the COVID-19 pandemic (8.5/100,000 in 2020 and 8.9/100,000 in 2021). This decrease can be seen across the EU/EEA during the pandemic, as according to the ECDC the IPD notification rate dropped from 5.8/100,000 in 2019 to 2.6/100,000 in 2020 [30]. In 2022, the IPD incidence in Slovenia again increased to 10.4/100,000. An increase in the IPD incidence after the COVID-19 pandemic has also been reported from England, and this is unsurprising given the results of this and other studies [31].

PCV-10 was introduced in the national vaccine recommendations programme in Slovenia in 2015, and in 2019 it was replaced by PCV-13. The introduction of pneumococcal vaccination in Slovenia influenced the incidence as well as serotype composition of IPD, but, as the vaccine uptake was not that high, the results were not so pronounced. The vaccine uptake in the period 2015–2019 had moderate coverage (49–65%), then in 2020 coverage increased to 70% of the target population, and in 2021 it fell to 58% [17]. The proportion of PCV13 serotypes in Slovenia decreased after the introduction of pneumococcal conjugate vaccine in the national programme, from 81.6% in 2015 to 45.5% in 2022. We recorded a decrease in the vaccine serotypes, especially serotypes 1, 14, 9V, 7F, 4 and 6A, and an increase in the serotypes 8, 22F, 11A, 6C, 23A and 15A, as well as an increase in serotype 3, which is present in PCV13. Several studies have confirmed the benefits of PCVs for preventing IPD caused by serotypes included in the vaccine in young children [32,33,34]. There are direct benefits of reduced sequelae among vaccinated children, as well as indirect benefits such as herd immunity and the reduced use of antimicrobial drugs. At the same time, limited serotype coverage of the vaccines has resulted in serotype replacement [34].

At the end of February 2022, most of the epidemiological measures due the COVID-19 pandemic had been lifted, so we could expect an increase in outpatient antibiotic use and consequently also an increase in the resistance of *S. pneumoniae*. It is therefore essential to continue national surveillance to evaluate the relaxation of interventions due to the pandemic, as well as the current vaccination programme and vaccines, and to continue to monitor both antibiotic consumption and microbial resistance in order to set appropriate antimicrobial guidelines.

## 4. Materials and Methods

### 4.1. Study Site

Slovenia is a small country with a population of roughly 2.1 million inhabitants, according to the 2022 census [35]. In Slovenia, virtually all residents (>99%) have mandatory basic health insurance, and a prescription is required for any type of antibiotic. In addition, only physicians can prescribe antibiotics for humans. Data on the number of antibiotic packages, the cost of antibiotics, the age and sex of patients and the identity and number of physicians and health care institutions prescribing antibiotics have been collected and published annually since 1976. In the period of 2015–2021, data on outpatient antibiotic use was collected using the ATC classification (ATC/defined daily doses (DDD)) [36]. The number of prescriptions for insured individuals and out-of-pocket paid antibiotics (“white” prescriptions for uninsured persons and prescriptions before travel) were provided by the National Institute of Public Health of Slovenia (NIPH) and Health Insurance Institute of Slovenia (HIIS).

### 4.2. Collection of Isolates and Serotyping

In Slovenia, the national surveillance of invasive diseases in children caused by *S. pneumoniae* has been continuously monitored since 1993, and in adults since 1996 [37]. All isolates of *S. pneumoniae* obtained from sterile body sites from patients with suspected invasive infection were collected from all Slovenian microbiological laboratories and sent to the Department for Public Health Microbiology, National Laboratory of Health, Environment and Food in Ljubljana. The isolates were identified by classical colony morphology and hemolysis on blood agar, and further tested for optochin susceptibility (Optochin Disc, Oxoid, UK) and bile solubility. A total of 2087 isolates of *S. pneumoniae* were collected from 2015 to 2022. All isolates were serotyped by Neufeld Quellung reaction using antisera provided by the Statens Serum Institut (Copenhagen, Denmark). For the purposes of the IPD resistance, we analysed the data from the period 2015 to 2022, and for the incidence the data from 2000 to 2022. A total of 2087 isolates of invasive *S. pneumoniae* were collected from 2015 to 2022 and a total of 5628 isolates of invasive *S. pneumoniae* were collected from 2000 to 2022.

### 4.3. Antibiotic Susceptibility Testing

Antibiotic susceptibility testing was performed using the disk diffusion method for oxacillin (screening test), erythromycin, clindamycin, vancomycin, and rifampicin. ETEST (bioMérieux, Marcy-l’Étoile, France) was performed to determine the minimum inhibitory concentrations (MIC) of penicillin, ampicillin, cefuroxime, cefotaxime, ceftriaxone, erythromycin, meropenem, tetracycline, chloramphenicol, trimethoprim/sulfamethoxazole, moxifloxacin, levofloxacin and linezolid. The recommendations of the European Committee on Antimicrobial Susceptibility Testing (EUCAST) were followed. *S. pneumoniae* ATCC 49619 isolate was used as the quality control [38]. Penicillin and erythromycin resistant strains refer to *S. pneumoniae* isolates that have MIC values to benzylpenicillin above 0.06 mg/L and MIC values to erythromycin above 0.5 mg/L. MIC data for co-resistance were analysed according to EUCAST 2022 (v 12.0) using the following breakpoints: benzylpenicillin R > 0.06, ampicillin R > 0.5, cefuroxime R > 1, cefotaxime R > 0.5, ceftriaxone R > 0.5, erythromycin R > 0.5, meropenem R > 0.25, tetracycline R > 2, chloramphenicol R > 8, trimethoprim-sulfamethoxazole R > 2, levofloxacin R > 2, moxifloxacin R > 0.5 and linezolid R > 2.

### 4.4. Data Analysis

Statistical analyses were performed using the free software R (R Core Team 2019, Vienna, Austria) [39]. Spearman’s rho2 rank correlation coefficients at a 95% confidence level were calculated to infer possible monotonic associations between *S. pneumoniae* resistance and total antibiotic and penicillin consumption expressed with DID (WHO versions of 2019) and RxIDs. To evaluate the impact of antibiotic consumption on the observed resistances more accurately, the latter were correlated with consumption from one year prior.

## 5. Conclusions

In Slovenia, the COVID-19 pandemic was associated with a substantial decrease in the total community consumption of antibiotics for systemic use. With a delay of one year, a decrease in the resistance of invasive *S. pneumoniae* isolates to penicillin and macrolides was also observed. The recommended national childhood pneumococcal vaccination, which was introduced in 2015, also had a substantial impact on the downward trend in incidence, resistance and decrease in vaccine serotypes in IPD in Slovenia from 2015 to 2022. After 2015, a downward trend in IPD incidence was observed, with a large decline during the COVID-19 pandemic (8.5/100,000 in 2020 and 8.9/100,000 in 2021). The proportion of PCV13 serotypes in Slovenia decreased from 81.6% in 2015 to 45.5% in 2022. In the observed period, two events took place that influenced the antibiotic consumption, incidence, resistance and predominant serotypes of invasive *S. pneumoniae* isolates in Slovenia. The reduced use of antibiotics was positively correlated with the reduced resistance of invasive pneumococci.

## Figures and Tables

**Table 1:** Community consumption of antibiotics for systemic use in Slovenia in the period 2015 and 2021.

Year	J01 DID	J01C DID	J01CR DID	J01FA DID	J01FA-FA1 DID	J01 RxIDs	J01C RxIDs	J01CR RxIDs	J01FA RxIDs	J01FA-FA1 RxIDs
2015	11.9	6.97	2.98	1.63	1.54	524.4	313.0	140.6	80.4	75.1
2016	11.5	6.79	2.83	1.36	1.28	494.4	299.2	133.4	66.3	62.1
2017	11.6	6.87	2.89	1.46	1.38	485.4	291.2	134.8	64.5	60.4
2018	11.7	6.87	2.85	1.55	1.46	480.6	285.9	133.3	64.7	60.0
2019	11.5	6.71	2.86	1.51	1.41	470.0	276.7	132.3	63.1	57.5
2020	8.8	4.7	2.36	1.22	1.17	357.6	191.6	118.9	47.5	45.1
2021	8.7	4.6	2.48	1.02	0.99	358.6	189.5	113.6	34.4	33.1

J01 antibiotics for systemic use, J01C penicillins, J01CR broad-spectrum penicillins, J01FA macrolides, J01FA-J01FA1 broad-spectrum macrolides, DID defined daily doses per 1000 inhabitants per day, RxIDs prescriptions per 1000 inhabitants per year.

**Table 2:** Resistance of invasive *Streptococcus pneumoniae* to penicillin (MIC > 0.06 mg/L) and macrolides (MIC > 0.5 mg/L) in Slovenia in 2015–2022.

Year	Resistance to Penicillin% (Number of Resistant Isolates/All)	Resistance to Macrolides % (Number of Resistant Isolates/All)
2015	9.1 (30/329)	18.8 (62/329)
2016	7.9 (22/279)	15.1 (42/279)
2017	10.1 (33/328)	13.4 (44/328)
2018	10.1 (28/276)	10.9 (30/276)
2019	12.3 (35/284)	9.9 (28/284)
2020	14.6 (26/178)	13.5 (24/178)
2021	6.3 (12/189)	7.4 (14/189)
2022	6.8 (15/220)	8.2 (18/220)

**Table 3:** Community consumption of antibiotics for systemic use (J01) and macrolides (J01FA) and resistance of *S. pneumoniae* to penicillin and macrolides in different countries in the period 2019–2021 [23,24,25,26,27].

Country (Antibiotic)	Consumption of Antibiotics (J01) and Macrolides (J01FA) in DID			Consumption (2019–2021)	Resistance of *S. pneumoniae* to Penicillin and Macrolides in %				Resistance (2019–2021)
	**2019**	**2020**	**2021**	**Δ%**		**2019**	**2020**	**2021**	**Δ%**
Slovenia (J01)	11.50	8.80	8.70	−24.4	Penicillin	12.30	14.60	6.30	−48.8
(J01FA)	1.51	1.22	1.02	−32.5	Macrolides	9.90	13.50	7.40	−25.3
Sweden (J01)	9.60	8.20	8.01	−16.6	Penicillin	6.90	8.30	6.30	−8.7
(J01FA)	0.25	0.25	0.22	−12.0	Macrolides	6.50	6.50	4.80	−26.2
Denmark (J01)	13.40	12.50	12.60	−6.0	Penicillin	4.90	7.20	10.10	+106.1
(J01FA)	1.41	1.15	1.11	−21.3	Macrolides	3.40	3.60	5.00	+47.0
The Netherlands (J01)	8.70	7.70	7.60	−12.7	Penicillin	4.00	4.80	6.20	+55.0
(J01FA)	1.22	1.13	1.07	−30.5	Macrolides	4.80	3.50	3.30	−31.3

## Data Availability

ESAC-Net, EARS-Net.
